# Trichostatin A and 5-azacytidine both cause an increase in global histone H4 acetylation and a decrease in global DNA and H3K9 methylation during mitosis in maize

**DOI:** 10.1186/1471-2229-10-178

**Published:** 2010-08-18

**Authors:** Fei Yang, Lu Zhang, Jun Li, Jing Huang, Ruoyu Wen, Lu Ma, Dongfeng Zhou, Lijia Li

**Affiliations:** 1 Key laboratory of MOE for Plant Developmental Biology, College of Life Sciences, Wuhan University, Wuhan 430072, China; 2 Tongji medical colleges, Huazhong Science and Technology University, Wuhan 430030, China

## Abstract

**Background:**

Modifications of DNA and histones in various combinations are correlated with many cellular processes. In this study, we investigated the possible relationship between histone H4 tetraacetylation, DNA methylation and histone H3 dimethylation at lysine 9 during mitosis in maize root meristems.

**Results:**

Treatment with trichostatin A, which inhibits histone deacetylases, resulted in increased histone H4 acetylation accompanied by the decondensation of interphase chromatin and a decrease in both global H3K9 dimethylation and DNA methylation during mitosis in maize root tip cells. These observations suggest that histone acetylation may affect DNA and histone methylation during mitosis. Treatment with 5-azacytidine, a cytosine analog that reduces DNA methylation, caused chromatin decondensation and mediated an increase in H4 acetylation, in addition to reduced DNA methylation and H3K9 dimethylation during interphase and mitosis. These results suggest that decreased DNA methylation causes a reduction in H3K9 dimethylation and an increase in H4 acetylation.

**Conclusions:**

The interchangeable effects of 5-azacytidine and trichostatin A on H4 acetylation, DNA methylation and H3K9 dimethylation indicate a mutually reinforcing action between histone acetylation, DNA methylation and histone methylation with respect to chromatin modification. Treatment with trichostatin A and 5-azacytidine treatment caused a decrease in the mitotic index, suggesting that H4 deacetylation and DNA and H3K9 methylation may contain the necessary information for triggering mitosis in maize root tips.

## Background

The basic unit of chromatin in eukaryotes is the nucleosome, which is composed of ~146 base pairs of DNA wrapped around an octameric core of the histone molecules H2A, H2B, H3 and H4 [1,2]. The amino-terminal tails of these histones are subject to various post-translational modifications such as methylation, acetylation, phosphorylation, ubiquitination and ADP-ribosylation [3]. Various histone-modifying enzymes able to add or remove chromatin modifications, including histone acetyltransferases (HATs), histone deacetylases (HDACs) and lysine methyltransferases, have been identified [3]. In yeast, HATs and HDACs have been found to alter global histone acetylation levels over large regions of chromatin [4]. DNA itself may be modified through the methylation of cytosine by DNA methyltransferase (DNMT) [5]. Histone and DNA modifications are dynamic and have wide-ranging and profound effects on many nuclear processes [6,7].

Epigenetic modifications dynamically alter chromatin structure and play an important role in the mitosis-dependent transition from decondensed interphase chromatin to condensed metaphase chromosome in eukaryotes. Phosphorylation of H3 is initiated in late G2, and this modification spreads along the chromatin as it undergoes condensation through the end of mitosis in mammalian cells [8] and plant cells [9,10]. The latest reports show that histone H3 phosphorylation at serine 10 (H3S10ph) is found in transcriptionally active regions such as the nucleolus [11]. In various mammalian cell lines, H4K5 is deacetylated in metaphase in contrast to interphase [8]. Mono- and diacetylation of newly synthesized histone H4 molecules are dramatically decreased from G2 to M phase in HeLa S3 cells [12]. In plants, the most intense H4 acetylation occurs during replication in both euchromatin and heterochromatin [13-15]. The deacetylation of H4 during the interphase to metaphase transition has been observed to be associated with chromatin condensation in tobacco protoplasts [10] as well as in barley [16,17]. In tobacco protoplasts, histone H3 dimethylation at lysine 9 (H3K9me2) and histone H3 dimethylation at lysine 4 (H3K4me2) levels remain unchanged during interphase and mitosis [10]. It has been reported that histone acetylation, histone methylation and DNA methylation are correlated and combined to regulate heterochromatin assembly in *Arabidopsis *[18]. However, little is known about the relationships among DNA methylation, histone methylation and acetylation during mitosis, from prophase to metaphase.

5-Azacytidine (5-AC), an analog of 5-cytosine, cannot be methylated and thereby inhibits DNA (5-cytosine) methylases, reducing the overall level of DNA methylation in chromatin [19-21]. Trichostatin A (TSA) is an inhibitor of HDACs and can be used to increase histone acetylation in chromatin [22]. TSA treatment of human fibroblasts for 12 hours was found to induce hyperacetylation of chromatin but did not prevent the progression of mitosis [8]. However, in human primary fibroblasts, TSA treatment resulted in impaired chromosome compaction and sister chromatid separation [23]. In addition, TSA treatment for 72 hours led to the accumulation of nuclei in metaphase and the appearance of abnormal anaphase events in tobacco protoplasts [10]. These observations imply that histone acetylation plays a complex role in the progression of mitosis.

To investigate the mitosis-dependent cross-talk between histone H4 tetraacetylation (H4ac), DNA methylation and H3K9 dimethylation (H3K9me2) as well as their influence on mitosis in maize, we examined chromatin conformational changes using a micrococcal nuclease (MNase) assay. We also used specific antibody immunostaining to detect and compare H4ac, H3K9me2 and DNA methylation patterns from prophase to metaphase following 5-AC or TSA treatment. We also analyzed the effect of 5-AC and TSA on mitotic index.

## Results

### TSA and 5-AC both induce changes in interphase chromatin conformation

**Figure 1:**
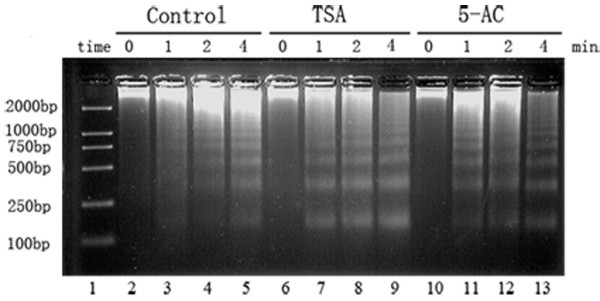
**MNase assay of control and TSA- and 5-AC-treated nuclei**. Chromatin from TSA- or 5-AC-treated nuclei after MNase digestion for various amounts of time (in minutes) shows increased sensitivity to MNase compared with chromatin from control nuclei. The left-most lane contains a DNA size marker.

To determine the chromatin state in TSA- and 5-AC-treated cells, Micrococcal nuclease (MNase) assays were carried out. As shown in Figure 1, after digestion with MNase, treated and untreated nuclei both yielded the typical nucleosome ladder, with a repeat length of about 170 bp. The chromatin from TSA-and 5-AC-treated nuclei was more sensitive to MNase digestion than chromatin from control nuclei (Figure 1), implying that TSA and 5-AC both induced chromatin decondensation.

### TSA and 5-AC both affect H4 acetylation, H3K9 dimethylation and DNA methylation

**Figure 2:**
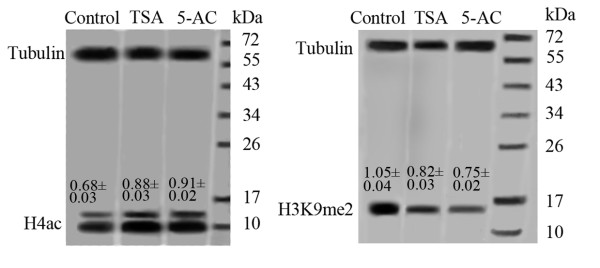
**Western blotting for H4ac (11 kDa) and H3K9me2 (17 kDa) in maize root tip cells treated with TSA or 5-AC**. Antibodies against tubulin were used as a protein loading control. Representative blots are shown. Average values of abundance index of H4ac and H3K9me2 were indicated in each lane (Mean ± SE). After treatment with either TSA or 5-AC, an increase in global H4ac and a decrease in global H3K9me2 were observed.

To assess the effects of TSA and 5-AC on levels of H4ac and H3K9me2, western blots were performed with anti-H4ac and anti-H3K9me2 antibodies. The results of the western blots showed that TSA caused a clear increase in global histone H4 acetylation (0.88 ± 0.03) in maize root tips compared with controls (0.68 ± 0.03). A decrease in total H3K9me2 was also detected in the TSA-treated nuclei (0.82 ± 0.03) compared with controls (1.05 ± 0.04) (Figure 2). Interestingly, 5-AC also caused a global increase in the level of H4ac (0.91 ± 0.02) and a decrease in the level of H3K9me2 (0.75 ± 0.02) (Figure 2).

**Figure 3:**
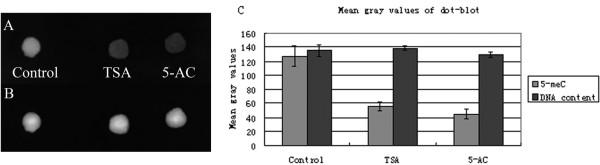
**Dot-blot for DNA methylation in maize root tip cells treated with TSA or 5-AC**. (A) Dot-blot analysis shows that 5-meC decreased in maize root tips after treatment with either 5-AC or TSA. (B) After immunodetection, the same membrane was stained with ethidium bromide to confirm equal DNA loading. (C) Mean gray values of dot-blots shown in (A) and (B). The mean gray value for 5-meC following treatment with either TSA or 5-AC was significantly lower than that of the control, while the mean gray value for DNA content in the control was not significantly different from that of the TSA- or 5-AC-treated sample. Error bars represent standard error of the mean.

A dot-blot immunoassay was performed on genomic DNA samples spotted on DEAE membranes to determine changes in overall DNA methylation levels in TSA- and 5-AC-treated cells (Figure 3). DNA was spotted onto a DEAE membrane, and anti-5meC antibody was used to detect cytosine methylation in the different DNA samples. Anti-5meC signals decreased after treatment with either TSA or 5-AC (Figure 3A), even though Figure 3B shows that the amount of each DNA sample bound to the membrane was approximately equal. The mean gray value for DNA methylation after treatment with TSA or 5-AC was significantly reduced compared with the value of the control by Student's t-test (P_TSA _= 0.0057 < 0.01; P_5-AC _= 0.0035 < 0.01). Moreover, the mean gray value for DNA content in the treated and untreated samples was not significantly different (P_TSA _= 0.3494 > 0.01; P_5-AC _= 0.2818 > 0.01) (Figure 3C).

Our results indicate that TSA and 5-AC both induced a change at the level of histone H4 acetylation, H3K9me2 methylation and DNA methylation, all of which are involved in chromatin decondensation in living maize root cells.

### TSA and 5-AC both affect cell cycle progression through mitosis in maize root meristems

To investigate the effects of these drugs on mitotic progression, the mitotic indices from each treatment condition were compared and three independent biological replicates were carried out. Student's t-test show that TSA and 5-AC treatments caused a significant decrease (P_TSA _= 0.007 < 0.01; P_5-AC _= 0.001 < 0.01) in the mitotic index (Table 1). After TSA- and 5-AC-treatment, the percentage of anaphase reduced from 0.873% to 0.424% (P_TSA _= 0.004 < 0.01) and from 0.873% to 0.280% (P_5-AC _= 0.004 < 0.01) respectively (Table 1) as well as the percentage of telophase significant decreased (P_TSA _= 0.01 ≤ 0.01; P_5-AC _= 0.004 < 0.01). These data indicate that TSA or 5-AC treatment leads to mitotic arrest and, therefore, the histone deacetylation and DNA methylation may play an important role in mitotic progression.

**Table 1:** Mitotic indices for control, TSA- and 5-AC-treated nuclei in maize root tips.

	*Control*		*TSA*		*5-AC*	
	**Mean**	**SE**	**Mean**	**SE**	**Mean**	**SE**

Mitosis	4.989%	0.098%	3.989%	0.023%	3.700%	0.101%
Prophase	2.376%	0.049%	1.822%	0.016%	1.823%	0.064%
Metaphase	1.152%	0.055%	1.462%	0.006%	1.378%	0.025%
Anaphase	0.873%	0.042%	0.424%	0.018%	0.280%	0.006%
Telophase	0.588%	0.033%	0.281%	0.006%	0.219%	0.011%
Total Number	5567.667	161.632	5815	93.675	5971	164.257

### Histone H4 is hyperacetylated in all stages of mitosis after treatment with either TSA or 5-AC

**Figure 4:**
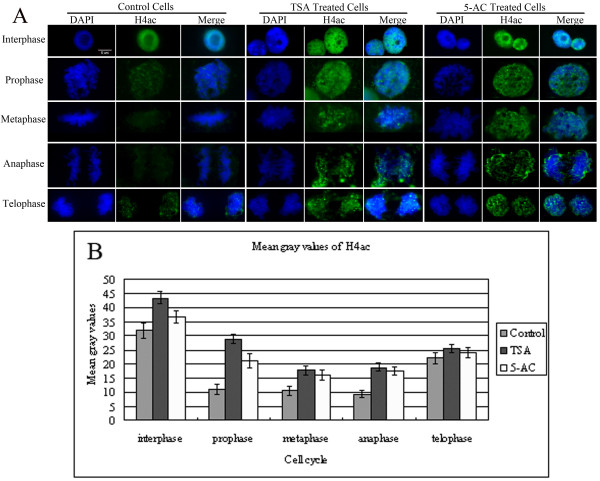
**Increased levels of H4ac in TSA- and 5-AC-treated cells**. (A) Immunostaining of H4ac in representative maize root tip cells in interphase or mitosis after TSA or 5-AC treatment. The 'DAPI' panel shows DAPI-stained DNA images, the 'H4ac' panel shows immunostained images and the 'Merge' panel shows the combined blue and green signals. In control cells, the strongly acetylated histone H4 signal was evenly distributed in the nucleus at interphase, and nucleoli were barely acetylated. At prophase, the deacetylation of H4 is initiated, and very weak acetylation is observed. As the cells progressed into metaphase and early telophase, the acetylated histone H4 signal is almost undetectable, whereas at telophase, H4 begins to be acetylated. In both TSA- and 5-AC-treated cells, strong hyperacetylation of histone H4 was detected in both mitotic and interphase cells of maize root tips compared with control cells. Bar = 5 μm. (B) Histogram showing mean gray values of the immunostaining signals for H4ac shown in (A). The mean gray value for H4ac after treatment with either TSA or 5-AC is higher than in the control. The mean gray values for H4ac were showed in Additional file 1: Table S1. Error bars represent the standard error of the mean.

To investigate in detail how H4 hyperacetylation affects mitosis, we performed *in situ *chromatin immunostaining for both normal maize root tip cells and TSA-treated cells. Deacetylation of histone H4 usually occurs during mitosis following chromatin condensation, as seen in the control cells. Immunostaining of normal interphase nuclei with anti-H4ac antibody showed that acetylated histone H4 was dispersed within the nucleus but that the nucleoli showed much less acetylation (Figure 4A). In control cells, the deacetylation of histone H4 was observed at prophase, and very weak acetylation signals were observed during metaphase and anaphase (Figure 4A). At telophase, anti-tetra-acetyl-histone H4 signals reappeared (Figure 4A). Figure 4A shows that TSA and 5-AC treatment resulted in the strong tetraacetylation of histone H4 during interphase and mitosis in maize root tip cells. Quantification of the signal intensity by measuring mean gray values showed that the acetylation of histone H4 was increased by approximately 10% to 50% after treatment with either TSA or 5-AC, compared with the control cells (Figure 4B).

### Treatment with either TSA or 5-AC causes a decrease in global H3K9me2 during interphase and mitosis

**Figure 5:**
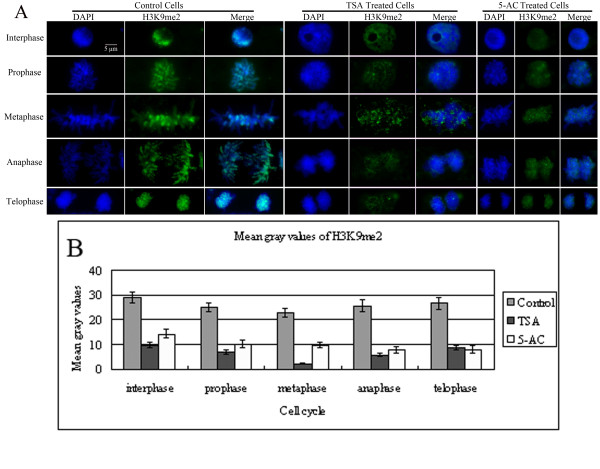
**Reduction in H3K9me2 in TSA- and 5-AC treated cells**. (A) Immunostaining for H3K9me2 in representative maize root tip cells in interphase or mitosis after TSA or 5-AC treatment. The 'DAPI' panel shows DAPI-stained DNA images, the 'H3K9me2' panel shows immunostained images and the 'Merge' panel shows a combination of blue and green signals. Dispersed H3K9me2 staining was observed throughout interphase and mitosis in the control cells. All TSA- and 5-AC-treated cells during interphase and mitosis have lower H3K9me2 levels than the untreated control cells. Bar = 5 μm. (B) Histogram showing mean gray values of the immunostaining signals for H3K9me2. The mean gray value for H3K9me2 after treatment with TSA or 5-AC is lower than that of the control. The mean gray values for H3K9me2 were derived from Additional file 1: Table S2. Error bars represent standard error of the mean.

Immunostaining for H3K9me2 showed dispersed labeling in interphase nuclei and nuclei from all phases of mitosis in control cells (Figure 5A). The mean gray values of the H3K9me2 signals revealed a subtle decrease in H3K9me2 following chromatin condensation. Furthermore, following chromatin decondensation, there was a slight increase in H3K9me2 during mitosis. Compared with untreated cells, a decrease in H3K9me2 was observed in interphase and at all stages of mitosis after treatment with either TSA or 5-AC (Figure 5A). Quantification of mean gray values showed that the H3K9me2 level was reduced by approximately 30% to 50% after TSA or 5-AC treatment (Figure 5B).

### TSA or 5-AC treatment causes a decrease in global DNA methylation during interphase and mitosis

**Figure 6:**
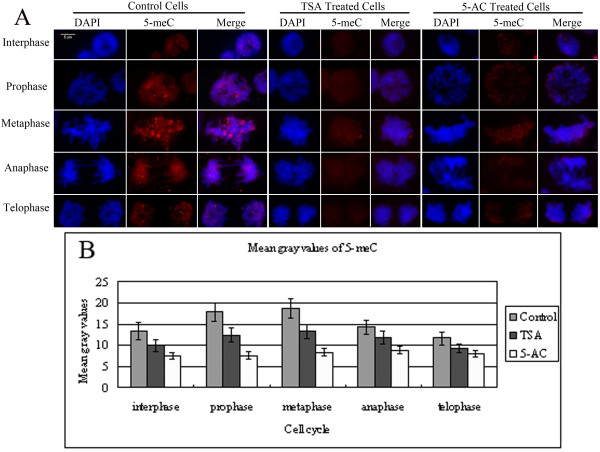
**Reduction in 5-meC in TSA- and 5-AC-treated cells**. (A) Immunostaining for 5-meC in representative maize root tip cells in interphase or mitosis after TSA or 5-AC treatment. The 'DAPI' panel shows DAPI-stained DNA images, the '5-meC' panel shows immunostained images and the 'Merge' panel shows a combination of blue and green signals. As the untreated control cells progressed into metaphase and early telophase, the amount of 5-meC within mitotic chromosomes increased compared with the amount in interphase cells. The DNA methylation level was reduced in each cell cycle phase after TSA and 5-AC treatments compared with control cells. Bar = 5 μm. (B) Histogram showing mean gray values of the immunostaining signals for 5-meC. The mean gray value of 5-meC signal after treatment with TSA or 5-AC is lower than that of the control. The mean gray values for 5-meC were derived from Additional file 1: Table S3. Error bars represent standard error of the mean.

Indirect immunostaining of 5-methylcytidine (5-meC) showed weak and dispersed DNA methylation within control nuclei. As the cells progressed into prophase and metaphase, the levels of 5-cytosine methylation after chromatin condensation during mitosis were gradually increased, and anti-5-meC signals began to decrease again at telophase (Figure 6A). TSA or 5-AC caused global DNA hypomethylation in both interphase and mitotic cells when the root tips were treated for 72 hours (Figure 6A). The histogram of mean gray values shows that DNA methylation was reduced by 10% to 20% after treatment with either TSA or 5-AC (Figure 6B).

## Discussion

### Dynamic changes in global H4ac, H3K9me2 and DNA methylation regulate chromatin state and affect mitotic progression in maize root tips

In our study, H4ac, H3K9me2 and DNA methylation showed cyclical alternations associated with chromatin condensation and decondensation during mitosis in maize root tip cells. Both TSA and 5-AC treatment induced (1) chromatin decondensation, (2) a reduction in the proportion of nuclei undergoing anaphase and telophase and (3) an increase in H4ac, a decrease in H3K9me2 and a decrease in DNA methylation during mitosis in maize root tips.

Posttranslational modifications contribute to driving changes in chromatin conformation and compaction [23]. H4K16ac has been found to inhibit the formation of compact 30-nanometer-like fibers as well as fiber-fiber interactions *in vitro *[24]. HDACs can function as part of chromatin-remodeling complexes [25]. Therefore, the inhibition of HDACs may influence the function of chromatin-remodeling complexes and subsequently affect chromatin condensation. 5-AC was reported to induce DNA hypomethylation, thus causing human chromosome decondensation [26]. DNA methylation prevents the histone octamer from interacting with an otherwise high-affinity positioning sequence [27] and thereby influences nucleosome formation [28].

Mitosis is a unique cell cycle phase in which duplicated chromosomes are highly condensed. During mitosis, dynamic changes in histone H4 acetylation accompanied by chromatin condensation/decondensation have been reported in mammalian, tobacco and barley cells [8,10,16,17]. In maize root tips, histone H4 deacetylation and reestablishing was also observed during mitosis. This mitosis-specific change in histone acetylation may be conserved in both mammalian and plant cells. In mammalian cells, H3K9me3 dramatically increases in G2, reaching a maximum at metaphase, and then rapidly declines during entry into the next interphase [29]. However, in tobacco protoplasts, there were no detectable changes in H3K9me2 and H3K4me2 throughout the cell cycle [10]. Our results show that, in maize root tips, cells entering mitosis with hypoacetylated histone H4 exhibit an altered chromatin conformation associated with H3K9me2 hypomethylation and DNA hypermethylation. Interestingly, changes in DNA methylation during mitosis were observed in maize root tips. The maize chromomethylase *Zea methyltransferase 2 *(*Zmet2*) has *de novo *activity and is responsible for the establishment of CpNpG methylation patterns [30]. Meanwhile, RNA-directed DNA methylation and demethylation provide plants with a versatile system that facilitates epigenetic plasticity during development and environmental stress responses [31]. The above mentioned pathways might both be involved in mitosis-specific DNA hypermethylation in maize.

In this study, we found that H4 hyperacetylation and DNA hypomethylation led to cell cycle arrest at metaphase associated with H3K9me2 hypomethylation in maize. ZmRpd3, a maize Rpd3-type histone deacetylase, can interact with the maize retinoblastoma-related (ZmRBR1) protein, an important regulator of cell cycle progression [32]. TSA, which inhibits the activity of histone deacetylases, might influence the reaction between ZmRpd3 and ZmRBR1 and alter cell cycle progression. H4 hyperacetylation arrests cells at metaphase or anaphase and causes abnormal anaphase in tobacco protoplasts [10]. In human cells, histone hyperacetylation can arrest cells in G2/M phase, prevent sister chromatin separation and cause chromosome segregation defects [23,33]. The HDAC3-AKAP95/HA95-Aurora B pathway regulating mitosis has been characterized in mammalian cells [34], and it is known that histone deacetylation is required for progression through mitosis in both plants and mammals. Complete inactivation of DNMT1 led to DNA hypomethylation and mitotic catastrophe in human cancer cells [35]. Our findings indicate that DNA methylation plays an important role in mitotic progression in maize root tips.

### Histone H4 hyperacetylation correlates with a decrease in H3K9me2 and DNA methylation

During both interphase and mitosis, we observed that inhibition of HDACs by TSA caused hyperacetylation of histone H4 and hypomethylation of H3K9 during mitosis in maize root tips. This observation suggests that the acetylation of chromatin proteins may affect histone methylation, either directly or indirectly. Earlier studies proposed that the methylation of lysine residues in histone tails functions as a static and irreversible epigenetic mark directing specific chromatin-mediated processes due to the absence of the corresponding histone demethylases [36]. However, two histone lysine demethylases, LSD1 and JmjC, have recently been identified to catalyze the removal of methyl groups on histone lysine residues [37,38]. The discovery of histone demethylases provides evidence for the dynamic nature of histone methylation. Our results raise the possibility that hyperacetylation of histone H4 recruits histone demethylases, resulting in the demethylation of H3K9. Histone hypoacetylation is often required to prepare the histone template for histone methyltransferases (HMTs) that act at different lysine residues [39].

We have determined that TSA mediates a reduction in the level of DNA methylation and an increase in histone acetylation in interphase and mitotic cells of maize root tips. Both DNA methylation and hypoacetylation of core histones are frequently associated with the repression of gene expression [40]. Analysis of DNA methylation demonstrated that TSA could cause selective loss of methylation in *Neurospora *[41]. Some reports have suggested that histone H4 hyperacetylation affects DNA methylation levels [42,43]. HATs and HDACs have been reported to alter global histone acetylation levels in yeast [4]. Although we cannot rule out the possibility that some gene sites did not lose their methylation, the TSA-mediated loss of global DNA methylation in interphase and mitotic cells that we observed in our experiments supports the conclusion that acetylation of histones can control DNA methylation [41].

### DNA hypomethylation leads to an increase in H4ac and a decrease in H3K9me2

The cytosine analog and DNA methylase inhibitor, 5-AC, is responsible for reducing DNA methylation. Genome-wide loss of DNA methylation was observed, and H4ac levels were increased in interphase and mitotic cells during maize root tip growth after 5-AC treatment, implying that DNA hypomethylation can also directly or indirectly affect histone acetylation. Methylated DNA sequences associated with epigenetic silencing in animals have also been found to be associated with hypoacetylated histones [44]. It is well established that DNA methylation can lead to the recruitment of HDACs [45]. The observation of interchangeable effects of 5-AC and TSA indicates a mutually reinforcing relationship between histone acetylation and DNA methylation in control of cellular processes.

H3K9me2 was also reduced during interphase and throughout mitosis after 5-AC treatment. There are several possible explanations for the histone hypomethylation effect. DNA hypomethylation may first cause histone hyperacetylation. Subsequently, as discussed above, hyperacetylation of histones would recruit histone demethylases that remove methyl groups on histone lysine residues [37,38]. Alternatively, DNA methylation may directly affect the extent of histone methylation. DNA methylation has been reported to be tightly correlated with histone H3 methylation [46]. Soppe *et al. *[18] demonstrated that DNA methylation controls the methylation of H3K9 and heterochromatin assembly in *Arabidopsis*, and this conclusion was supported by Tariq *et al. *[47].

## Conclusions

**Figure 7:**
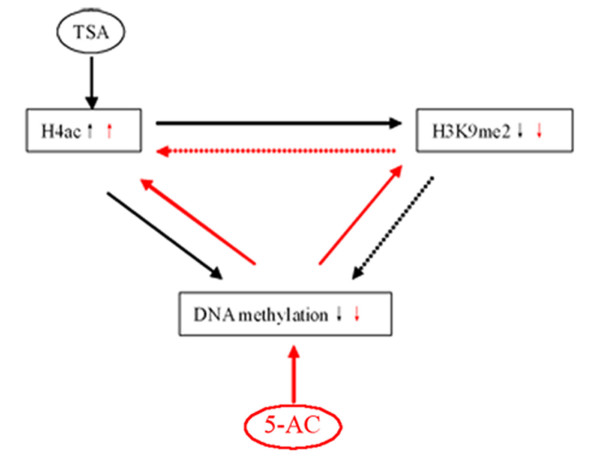
**Model depicting cross-talk between histone H4 acetylation, DNA methylation and H3K9me2**. The black arrow shows the TSA-mediated pathway, and the red arrow displays the 5-AC-mediated pathway. TSA induces an increase in H4ac, which subsequently causes a decrease in H3K9me2 and DNA methylation. 5-AC causes a decrease in DNA methylation, which is followed by an increase in H4ac and a decrease in H3K9me2. DNA methylation and H4ac both precede and control H3K9me2, while H4ac and DNA methylation affect each other. H3K9me2 could also feed back on H4 acetylation or/and DNA methylation (dashed red and black arrows).

Therefore, we propose that a mutually reinforcing cross-talk exists between histone acetylation, DNA methylation and H3K9me2 (Figure 7). Histone H4 hyperacetylation may cause a loss of global DNA methylation, and DNA hypomethylation may mediate histone H4 acetylation. Histone H4 hyperacetylation and DNA hypomethylation may mediate decreased H3K9me2 levels, but it is also possible that a reduction in H3K9me2 may feed back onto the extent of H4ac and DNA methylation. This model needs to be further modified or refined and supplemented using histone methylation mutants or inhibitors and other experimental systems. More studies will be required to discover the cross-talk connections between modifications of histones and DNA and between these modifications and cellular functions.

## Methods

### Plant material and antibodies

Seeds from the *Zea mays *L. inbred line Huangzao 4 [48] were kindly provided by Prof. Song Jiancheng, Shandong Agriculture University, Shandong Province, China. Seeds were germinated for 3 days at 25°C either on filter paper soaked in water or in water containing 15 μM TSA or 80 μM 5-AC [49] in culture tanks. For each treatment as well as the control, three independent replicate experiments were carried out.

The primary antibodies used in this study were rabbit anti-tetra-acetyl-histone H4 (anti-H4ac) (06-866, Upstate, USA), rabbit anti-dimethyl-histone H3 Lys9 (anti-H3K9me2) (07-441, Upstate, USA), rabbit anti-α-tubulin (05-829, Upstate, USA) and mouse anti-5-methylcytidine (anti-5meC) (12-507, Serotec, UK). The secondary antibodies were fluorescein-conjugated goat anti-rabbit IgG (12-507, Millipore, USA), Cy3-conjugated goat anti-mouse IgG (AP124C, Millipore, USA) and alkaline phosphatase (AP)-conjugated goat anti-rabbit IgG (S3731, Promega, USA).

### Micrococcal nuclease assay

Micrococcal nuclease (MNase) assays were performed as described by Zhao *et al. *[50] with some modifications. Nuclei were prepared from control, TSA- or 5-AC-treated maize root tips as previously described [51]. Equal amounts of prepared nuclei were washed and resuspended in 1 ml of nuclear digestion buffer (50 mM Tris-HCl, pH 8.0, 0.1 mM CaCl_2_). MNase (2000 units/ml, Sigma) was then added, and digestions were carried out for different durations. Each sample was extracted once with phenol/chloroform/isoamyl alcohol (25:24:1). Finally, the MNase digestion products were resolved on 2% agarose gels and stained with ethidium bromide. Three independent replicate experiments were carried out.

### Western blotting

All procedures were performed as described by Luo *et al. *[52]. Briefly, about 400 mg of maize root tips were homogenized using 400 μl of protein extraction buffer (66 mM Tris, pH 6.8, 2% sodium dodecyl sulfate (SDS), 1 mM dithiothreitol (DTT) followed by centrifugation at 10,000 g for 20 min at 4°C. The protein concentration of the supernatant was determined using a DC Protein Assay Kit II (Bio-Rad, CA). About 10 μg of protein in 10 μl of sample buffer (50 mM Tris, pH 6.8, 100 mM DTT, 2% SDS, 0.1% bromophenol blue, 10% glycerol) was loaded onto a 12% polyacrylamide gel and separated by electrophoresis. Samples were then transferred to a nitrocellulose membrane according to the manufacturer's instructions (Bio-Rad, CA). Anti-H4ac and anti-H3K9me2 antibodies (Upstate, USA) (1:1000) were used for western blotting. The anti-α-tubulin antibody (Upstate, USA) (1:1000) was used as control of constitutive protein expression. The secondary antibody used was AP-conjugated goat anti-rabbit IgG (Promega, USA) (1:1000) and revealed by treatment with a nitroblue tetrazolium, bromo-chloro-indolyl-phosphate (NBT-BCIP) mixture. Densitometric measurements were taken after immunodetection using the Alphalmager TM 2200 System (Alpha Innotech Corporation, USA). Abundance index was calculated as follows: H4Ac or H3K9me2 band intensity/α-tubulin band intensity. Three independent replicate experiments were carried out. Mean abundance index and standard error of the mean were calculated with SPSS10.0 for Windows package (SPSS Inc., 1999).

### Dot-blot analysis of DNA methylation levels

Dot-blot analysis of DNA methylation was performed according to the method reported by Oakeley *et al. *[53]. Three micrograms of DNA from treated and untreated samples was spotted onto a DEAE membrane (Schleicher & Schuell, NA 85) and incubated with anti-5meC antibody (1:1000) for 16 h at 4°C. The membrane was washed with DEAE wash buffer (50 mM NaCl, 10 mM Tris-HCl, 1 mM EDTA, pH 7.5, 1% Triton X-100) for 30 min at room temperature and then incubated overnight with a Cy3-conjugated goat anti-mouse IgG secondary antibody (1:1000). Signals were detected using a Storm PhosphorImager (Molecular Dynamics, USA). After detection, the membrane was stained for 30 sec in DEAE wash buffer containing 500 μg/ml ethidium bromide. Next, the membrane was destained for 0.5 h with DEAE wash buffer. The DNA was then visualized using a UV transilluminator. Dot-blots were repeated three times using samples from independent experiments. Mean gray values of each dot were calculated with Image J. Three independent replicate experiments were carried out. Independent t test analysis was performed with SPSS10.0 for Windows package (SPSS Inc., 1999).

### Measuring mitotic index

For each treatment, at least 5000 nuclei were randomly selected for calculation of the mitotic index and the percentage of nuclei in each phase of mitosis. In these samples, nuclei in each phase of mitosis were identified and counted. The mitotic index was defined as the ratio between the number of mitotic nuclei and the number of total nuclei. The percentage of nuclei in each mitotic phase was determined by counting the number of nuclei found in each phase in a sample population including both interphase and mitotic nuclei. This analysis was carried out for three biological replicates. Independent t test analysis was performed with SPSS10.0 for Windows package (SPSS Inc., 1999).

### Immunostaining

Root tips were excised, fixed in freshly prepared 4% (w/v) paraformaldehyde in 1× phosphate-buffered saline (PBS; 0.137 M NaCl, 2.7 mM KCl, 1.4 mM KH_2_PO_4_, 4.3 mM Na_2_HPO_4_, pH 7.3) for 20 min at 4°C and then washed with 1× PBS for 10 min. Subsequently, root tips were treated with a mixture of 2% cellulase and 2% pectolyase dissolved in 1× PBS for 50-70 min at 37°C. Two or three root tips were squeezed into a drop of 1× PBS on a slide, and a cover slip was added. Chromosomes were squashed and spread by applying gentle pressure to the cover slip. The cover slip was subsequently removed with a razor blade after the slide was frozen in liquid nitrogen [15].

The prepared slides were incubated for 1 h at 37°C in 3% BSA dissolved in 1× PBS, washed for 15 min with 1× PBS and finally incubated overnight at 4°C with anti-H4ac, anti-H3K9me2 or anti-5meC. Each antibody was diluted 1:500 in 3% BSA dissolved in 1× PBS. The slides were washed twice with 1× PBS for 5 min and incubated for 1 h with the appropriate secondary antibody (fluorescein-conjugated goat anti-rabbit IgG or Cy3-conjugated goat anti-mouse IgG). Secondary antibodies were diluted 1:500 in 3% BSA. Immunostained slides were washed three times for 5 min with 1× PBS. In control experiments, slides were incubated with the secondary antibody alone. All slides were counterstained with 0.2 μg/ml DAPI (4',6-diamidino-2-phenylindole, Sigma, USA), mounted with Vectashield (Vector labs, USA) and examined on an Olympus BX-60 fluorescence microscope with filter blocks for Cy3, DAPI and fluorescein. Images captured with a CCD monochrome camera Sensys 1401E were pseudo-colored and merged using MetaMorph^® ^4.6.3 software (Universal Imaging Corp., USA). Microscope settings and camera detector exposure times were kept constant for each respective channel (Cy3, fluorescein, or DAPI) but were optimized for individual experiments. All images were processed using Adobe Photoshop 9.0 software. The phase of mitosis was identified in each nucleus, and the mean gray value of the signal intensity was measured with Image J and MetaMorph. Three independent replicate experiments were carried out. Mean gray value of the signal intensity and standard error of the mean were calculated with SPSS10.0 for Windows package (SPSS Inc., 1999).

## Authors' contributions

FY conceived the study, carried out the experiments, performed the statistical analysis and drafted the manuscript. LZ participated in the immunostaining. JL participated in the molecular studies and helped to draft the manuscript. JH participated in the immunostaining. RW, LM and DZ helped to draft the manuscript. LL conceived and designed the study and drafted the manuscript. All authors read and approved the final manuscript.

## Supplementary Material

Additional file 1 **Mean gray value of epigenetic modifications in cell cycle**. Table S1 - Mean gray value of H4ac in cell cycle. Table S2 - Mean gray value of H3K9me2 in cell cycle. Table S3 - Mean gray value of DNA methylation in cell cycle. Click here for file
