# Supplementary material for: Trichostatin A and 5-azacytidine both cause an increase in global histone H4 acetylation and a decrease in global DNA and H3K9 methylation during mitosis in maize
Source: BMC Plant Biol. 2010 Aug 18;10:178. doi: 10.1186/1471-2229-10-178 (PMC3095308; doi:10.1186/1471-2229-10-178)
Supplement: Additional file 1 — Mean gray value of epigenetic modifications in cell cycle. Table S1 - Mean gray value of H4ac in cell cycle. Table S2 - Mean gray value of H3K9me2 in cell cycle. Table S3 - Mean gray value of DNA methylation in cell cycle. [file 1471-2229-10-178-S1.DOC]

# Additional files

## Additional file 1-Mean gray value of epigenetic modifications in cell cycle.

Table S1 – Mean gray value of H4ac in cell cycle.

|  | **Control** | | **TSA** | | **5-AC** | |
| --- | --- | --- | --- | --- | --- | --- |
| **Cell cycle phase** | **Mean nuclei number** | **Mean gray value** | **Mean nuclei number** | **Mean gray value** | **Mean nuclei number** | **Mean gray value** |
| **interphase** | **200±0** | **31.735±2.621** | **200±0** | **43.459±2.174** | **200±0** | **36.628±2.385** |
| **prophase** | **39.667±0.882** | **11.041±1.876** | **37.333±2.0276** | **28.758±1.689** | **37.333±1.764** | **21.172±2.374** |
| **metaphase** | **24.333±0.882** | **10.743±1.556** | **25.000±2.309** | **17.588±1.759** | **24.333±0.882** | **15.901±1.819** |
| **anaphase** | **18.333±1.764** | **9.249±1.334** | **17.000±0.577** | **18.805±1.354** | **16.667±0.882** | **17.488±1.364** |
| **telophase** | **14.333±1.202** | **22.036±2.147** | **13.667±0.333** | **25.584±1.357** | **14.000±0.577** | **23.761±1.804** |

Table S2 – Mean gray value of H3K9me2 in cell cycle.

|  | **Control** | | **TSA** | | **5-AC** | |
| --- | --- | --- | --- | --- | --- | --- |
| **Cell cycle phase** | **Mean nuclei number** | **Mean gray value** | **Mean nuclei number** | **Mean gray value** | **Mean nuclei number** | **Mean gray value** |
| **interphase** | **200±0** | **28.751±2.132** | **200±0** | **9.798±0.937** | **200±0** | **14.214±1.851** |
| **prophase** | **41±1.528** | **24.973±1.965** | **38.667±1.201** | **6.786±0.987** | **37.333±1.453** | **10.129±1.728** |
| **metaphase** | **21.333±1.202** | **22.727±1.892** | **20.667±0.882** | **2.181±0.345** | **25.333±1.453** | **9.656±1.352** |
| **anaphase** | **16.333±0.882** | **25.445±2.354** | **17.000±0.577** | **5.643±0.542** | **16.667±0.882** | **7.762±1.415** |
| **telophase** | **14.667±1.667** | **26.511±2.439** | **14.333±0.882** | **8.786±0.819** | **14.000±1.000** | **7.899±1.498** |

Table S3 – Mean gray value of DNA methylation in cell cycle.

|  | **Control** | | **TSA** | | **5-AC** | |
| --- | --- | --- | --- | --- | --- | --- |
| **Cell cycle phase** | **Mean nuclei number** | **Mean gray value** | **Mean nuclei number** | **Mean gray value** | **Mean nuclei number** | **Mean gray value** |
| **interphase** | **200±0** | **13.371±1.985** | **200±0** | **9.921±1.392** | **200±0** | **7.551±0.797** |
| **prophase** | **40.000±1.732** | **17.772±2.198** | **39.000±1.155** | **12.445±1.673** | **37.333±1.453** | **7.641±0.841** |
| **metaphase** | **25.333±1.202** | **18.673±2.339** | **24.667±1.856** | **13.235±1.671** | **25.667±1.453** | **8.252±0.958** |
| **anaphase** | **16.667±0.882** | **14.173±1.582** | **18.000±0.577** | **11.797±1.539** | **16.667±0.882** | **8.819±0.839** |
| **telophase** | **14.667±1.764** | **11.615±1.548** | **14.667±0.882** | **9.254±0.949** | **15±1.732** | **7.998±0.851** |
